# First report from the Czech national registry of inborn errors of immunity (2012–2025)

**DOI:** 10.3389/fimmu.2025.1653685

**Published:** 2025-09-15

**Authors:** Zita Chovancova, Eva Hlavackova, Roman Hakl, Tomas Milota, Pavlina Kralickova, Ivana Malkusova, Beata Hutyrova, Michaela Safarova, Jana Vydlakova, Dalibor Jilek, Jiri Novak, Helena Schneiderova, Petra Kralova, Alena Zimulova, Vitezslav Novak, Jaromir Bystron, Dita Zaveska, Vendula Latalova, Frantisek Kopriva, Milan Kasl, Vladimir Kracik, Renata Formankova, Petr Sedlacek, Karolina Vytiskova, Michal Svoboda, Hana Novakova, Jiri Litzman

**Affiliations:** ^1^ Department of Clinical Immunology and Allergology, St. Anne´s University Hospital in Brno, Brno, Czechia; ^2^ Faculty of Medicine, Masaryk University, Brno, Czechia; ^3^ Department of Immunology, Second Faculty of Medicine Charles University and University Hospital in Motol, Prague, Czechia; ^4^ Institute of Clinical Immunology and Allergy, University Hospital, Hradec Kralove, Czechia; ^5^ Faculty of Medicine in Hradec Kralove, Charles University, Hradec Kralove, Czechia; ^6^ Department of Allergology and Clinical Immunology, University Hospital Pilsen, Pilsen, Czechia; ^7^ Department of Allergology and Clinical Immunology, University Hospital Olomouc, Olomouc, Czechia; ^8^ Institute of Immunology and Microbiology of the First Faculty of Medicine and General University Hospital, Prague, Czechia; ^9^ Department of Clinical Immunology and Allergology, Institute for Clinical and Experimental Medicine, Prague, Czechia; ^10^ Department of Clinical Immunology and Allergology, Masaryk Hospital, Usti nad Labem, Czechia; ^11^ Centre for Clinical Immunology, Hospital Ceske Budejovice, Ceske Budejovice, Czechia; ^12^ Department of Pediatrics, University Hospital Brno, Brno, Czechia; ^13^ Department of Allergology and Clinical Immunology, Faculty Hospital Kralovske Vinohrady, Prague, Czechia; ^14^ Department of Pulmonary Medicine, Tomas Bata Regional Hospital, Zlin, Czechia; ^15^ Department of Immunology and Allergy, Public Health Institute Ostrava, Ostrava, Czechia; ^16^ Department of Allergology and Clinical Immunology, Faculty Hospital Ostrava, Ostrava, Czechia; ^17^ Department of Pediatrics, University Hospital and Faculty of Medicine in Hradec Kralove, Charles University, Hradec Kralove, Czechia; ^18^ Department of Pediatrics, Faculty of Medicine and Dentistry, Palacky University, Olomouc, Czechia; ^19^ Kasmed Ltd., Tabor, Czechia; ^20^ Department of Clinical Microbiology and Immunology, Liberec Regional Hospital, Liberec, Czechia; ^21^ Department of Pediatric Hematology and Oncology, Second Faculty of Medicine, Charles University and Motol University Hospital, Prague, Czechia; ^22^ Institute of Biostatistics and Analyses, Ltd., Brno, Czechia

**Keywords:** registry report, primary immunodeficiency, inborn errors of immunity, Czech national registry, hematopoietic stem cell transplantation, immunoglobulin replacement therapy

## Abstract

**Introduction:**

Congenital immune system defects represent an ever-growing group of diseases characterized by increased susceptibility to infections and association with autoimmune, autoinflammatory, allergic and malignant complications. Here, we provide the first comprehensive report on inborn errors of immunity (IEIs) in Czechia based on the analysis of patient data from the Czech national registry (CzNR) of IEIs.

**Material and methods:**

The online platform of CzNR of IEIs was established in 2012, compiling data about epidemiology, type of diagnosis, clinical and laboratory parameters, as well as the treatment of patients diagnosed with IEIs since 1981.

**Results:**

The total of 1,443 registered patients includes 697 males (48.3%) and 746 females (51.7%). The median age at diagnosis was 21.0 (0–86) years. The most represented group of patients was those with antibody deficiencies (788 patients; 54.6%). This was followed by complement deficiencies (242; 16.8%), combined immunodeficiencies with syndromic features (250; 17.3%), combined immunodeficiencies (55; 3.8%), congenital defects of phagocyte number, function, or both (31; 2.1%), autoinflammatory disorders (28; 1.9%), immune dysregulation diseases (24; 1.7%), intrinsic and innate immunity defects (21; 1.5%), primary immunodeficiency phenocopies (3; 0.2%), and bone marrow failure disorders (1; 0.1%). Common variable immunodeficiency (504; 34.9%), hereditary angioedema (222; 15.4%), and DiGeorge syndrome (182; 12.6%) were the most frequent diagnoses.

**Conclusion:**

In this article, we report the epidemiology of IEIs in the Czech Republic for the first time based on the CzNR of IEIs data. The prevalence of IEIs is approximately 13.2 patients per 100000 inhabitants of the Czech Republic.

## 1 Introduction

Congenital immune system disorders affecting innate and adaptive immune system mechanisms have been recognized for more than 70 years. In 1952, pediatrician Ogden Carr Bruton described the first case of what is now a well-defined group of diseases (1). This marked the beginning of a long journey in the study of disorders that impair immune system function. Initially, this group of diseases was referred to as “primary immunodeficiencies” (PIDs) due to their congenital genetic origin and predominant symptom of increased susceptibility to infections. Over time, it became evident that their clinical manifestation also frequently includes autoimmune, autoinflammatory, allergic, and malignant complications. As a result, the term “inborn errors of immunity” (IEIs) has come into use, better reflecting the broader spectrum of immune dysregulation (2).

The first classification of PID was proposed in 1970 by a World Health Organization (WHO) Expert Committee. The initial report identified 16 distinct immunodeficiencies, classified as either B-cell or T-cell disorders (3). Today, IEIs represent a group of over 500 rare monogenic immune system disorders, a number that continues to grow (2). Although they are considered rare diseases, their true prevalence is likely underestimated. Patient registries, especially for rare conditions, have proven to be essential tools for evaluating the clinical, epidemiological, and therapeutic characteristics of affected individuals. The first national registries of congenital immune system disorders were established in various countries during the early 1980s (4–16). Over time, larger international collaborative networks have emerged to integrate data from multiple countries and regions—for example, the European Society for Immunodeficiencies (ESID) registry in Europe, the United States Immunodeficiency Network (USIDNET) databases in the USA, and the Latin American Society for Immunodeficiencies (LASID) in Latin America (17–19).

In the Czech Republic, the first efforts to monitor patients with PID began in 1981. These laid the groundwork for the establishment of the Czech National Registry of Primary Immunodeficiencies (CzNR) in 1995, intended to collect epidemiological data on affected individuals. However, the data from this early registry were never formally published. In 2012, the online CzNR of IEIs was launched as a non-interventional clinical study. The first patient with a diagnosis of common variable immunodeficiency (CVID) was entered into the registry on 13th April 2012. In this article, we present, for the first time, the epidemiological data of IEIs in the Czech Republic, based on records from the CzNR of IEIs.

## 2 Materials and methods

### 2.1 Registry structure and patient characteristics

Patients were diagnosed according to ESID diagnostic criteria (20, 21). Individuals with secondary immune deficiencies were excluded from the registry. Basic demographic data collected included date of birth, sex, patient initials, date of informed consent, ESID registry number, type of health insurance, district of residence, and date of diagnosis. Patients were categorized in the registry according to their IEIs diagnosis into 10 groups based on the International Union of Immunological Societies (IUIS) classification: predominantly antibody deficiencies, complement deficiencies, combined immunodeficiencies with syndromic features, combined immunodeficiencies, congenital defects of phagocyte number, function, or both, autoinflammatory disorders, immune dysregulation diseases, intrinsic and innate immunity defects, PID phenocopies, and bone marrow failure disorders (2). Patients with unclassified diagnoses were assigned to the “unknown PID” group. Collected treatment data included hematopoietic stem cell transplantation (HSCT), immunoglobulin replacement therapy (IRT), antibiotic prophylaxis, immunosuppressive treatment, splenectomy, and psychiatric medication (primarily clozapine). A separate section was dedicated to the treatment of hereditary angioedema (HAE) attacks. Patients with multiple overlapping phenotypes were entered into the registry according to their primary diagnosis based on the IUIS classification, even if they suffered from additional associated immunopathological complications.

### 2.2 Centers

The Czech Republic, located in Central Europe, has nearly 11 million inhabitants and an area of 78,866 km^2^. It is divided into 14 self-governing regions. Almost all regions have medical facilities that provide care for patients with IEIs and contribute data to the CzNR of IEIs. Participating medical centers include: Prague (Department of Immunology, Second Faculty of Medicine and University Hospital in Motol, Charles University; Institute of Immunology and Microbiology of the First Faculty of Medicine and General University Hospital; Department of Clinical Immunology and Allergology, Institute for Clinical and Experimental Medicine; Department of Pediatric Hematology and Oncology, Second Faculty of Medicine, Charles University and Motol University Hospital, and Department of Allergology and Clinical Immunology, Faculty Hospital Kralovske Vinohrady), South Bohemian Region (Centre for Clinical Immunology, Hospital Ceske Budejovice; Kasmed Ltd. in Tabor), Plzen Region (Department of Allergology and Clinical Immunology, University Hospital Pilsen), Usti nad Labem Region (Department of Clinical Immunology and Allergology, Masaryk Hospital Usti nad Labem), Liberec Region (Department of Clinical Microbiology and Immunology, Liberec Regional Hospital), Hradec Kralove Region (Institute of Clinical Immunology and Allergology and Department of Pediatrics, University Hospital and Faculty of Medicine in Hradec Kralove, Charles University), South Moravian Region (Department of Clinical Immunology and Allergy, St. Anne´s University Hospital in Brno; Department of Pediatrics, University Hospital Brno), Olomouc Region (Department of Allergology and Clinical Immunology, University Hospital Olomouc; Department of Pediatrics, Faculty of Medicine and Dentistry, Palacky University), Zlin Region (Department of Pulmonary Medicine, Tomas Bata Regional Hospital Zlin) and Moravian-Silesian Region (Department of Immunology and Allergy, Public Health Institute Ostrava; Department of Allergology and Clinical Immunology, Faculty Hospital Ostrava). No designated centers for IEIs care were available in the Central Bohemian, Karlovy Vary, Pardubice, and Vysocina regions.

### 2.3 Registry platform

Clinical Data Warehousing Information System (CLADE-IS) is an electronic data capture (EDC) system developed by the Institute of Biostatistics and Analyses (IBA). CLADE-IS operates in most standard web browsers, eliminating the need for additional software installation, and offers a user-friendly and ergonomic interface. The system includes built-in protections against Structured Query Language (SQL) injection and cross-site scripting. Only authorized users with assigned roles can access the system via secure login credentials. Data entry is performed by authorized healthcare professionals (physicians, nurses) or designated hospital data managers. All patient data are pseudonymized, with each patient assigned a unique identifier.

### 2.4 Data verification and validation

All data entered into CLADE-IS undergo multiple validation steps. The system includes pre-programmed validation rules that ensure data quality by checking value ranges, logical consistency, and inter-field dependencies. These checks occur automatically during data entry and save operations and do not require user activation. Newly enrolled patients are screened for potential duplicates using date of birth and sex. If a match is detected, the system prompts the user to confirm whether the patient is already registered. In cases of suspected duplicates across different sites, the Helpdesk provides support. When a patient changes healthcare provider, their data may be transferred to the new site upon request. Based on mutual agreement, both the original and new providers may retain access to the patient’s record if clinically necessary.

Data collection and processing in the EDC system comply with IBA’s internal quality management procedures and adhere to the standards of EN ISO 9001:2015 (Quality Management), ISO/IEC 27001:2022 (Information Security Management), and Good Clinical Practice. Additionally, the system is compliant with EMA guidelines on computerized systems and electronic data in clinical trials.

### 2.5 Statistical analysis

No formal statistical hypothesis was defined; the analysis was purely descriptive. Categorical variables were summarized using absolute and relative frequencies. Continuous variables were described using mean ± standard deviation and median (range). The number of patients per 100,000 inhabitants in each district was calculated using population data from the Czech Statistical Office (https://csu.gov.cz/).

## 3 Results

**Figure 1:**
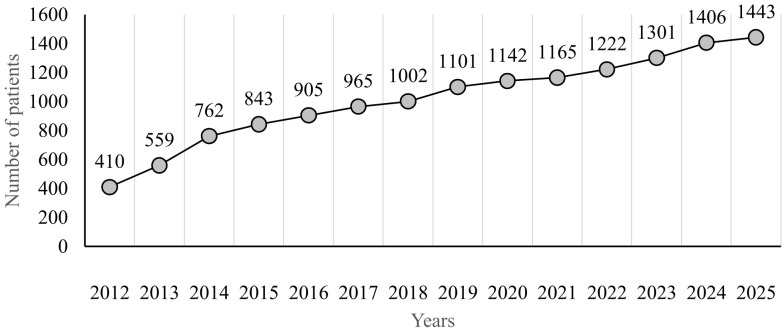
Rate of data accrual by 18 centres from 2012−2025.

The total number of registered patients in the period from 13^th^ April 2012 to 10^th^ February 2025 was 1,443. Of these, 1,173 (81.3%) were actively followed, while 119 (8.2%) were deceased, 98 (6.8%) were lost to follow-up, and 53 (3.7%) had their monitoring discontinued for other reasons. Of all patients, 697 (48.3%) were males and 746 (51.7%) were females. The rate of data accrual is shown in **Figure 1**.

### 3.1 Age distribution of patients

**Figure 2:**
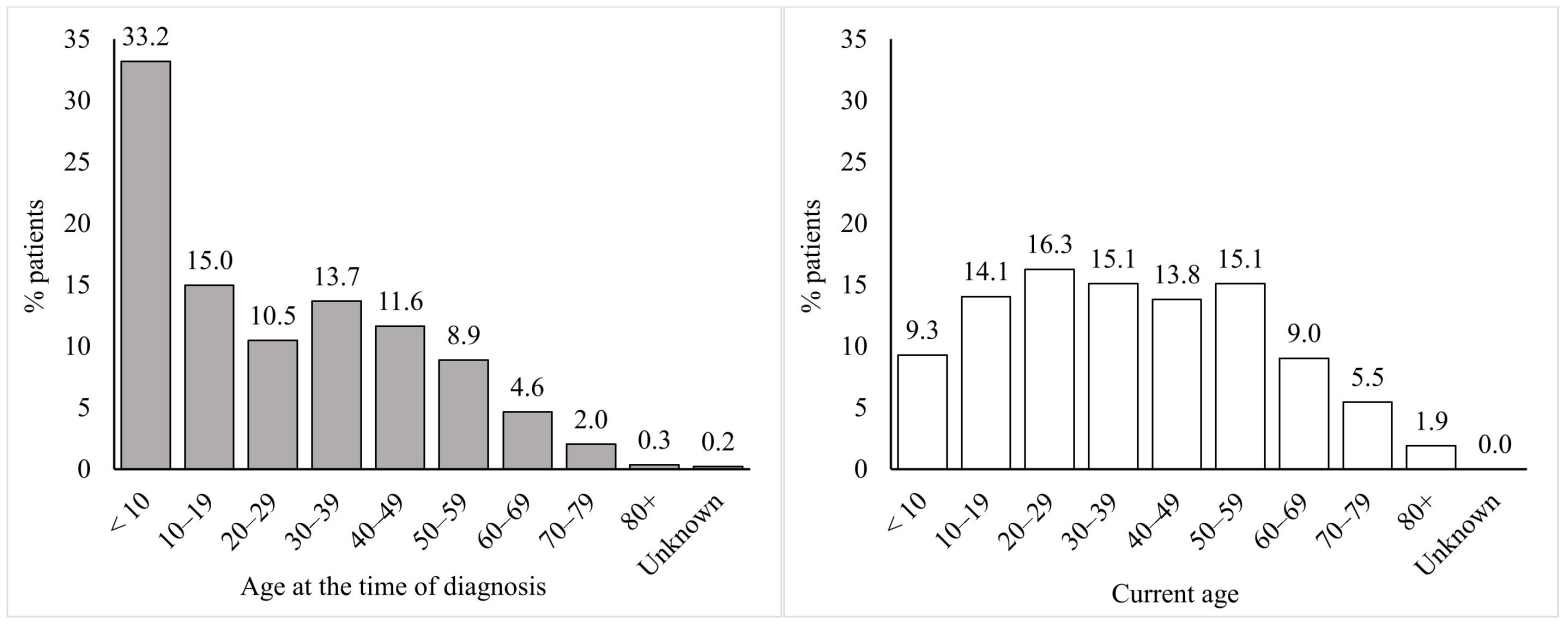
Age distribution of patients in the Czech National Registry.

Out of 1,443 registered patients, the mean age at diagnosis was 24.9 ± 21.2 years (median: 21 years; range: 0–86). In the subgroup of 1,173 followed patients, the current age was 37.5 ± 20.9 years (median: 37 years; range 1–91); see **Figure 2**.

### 3.2 Representation of patients by IEI diagnostic groups according to the IUIS classification

**Figure 3:**
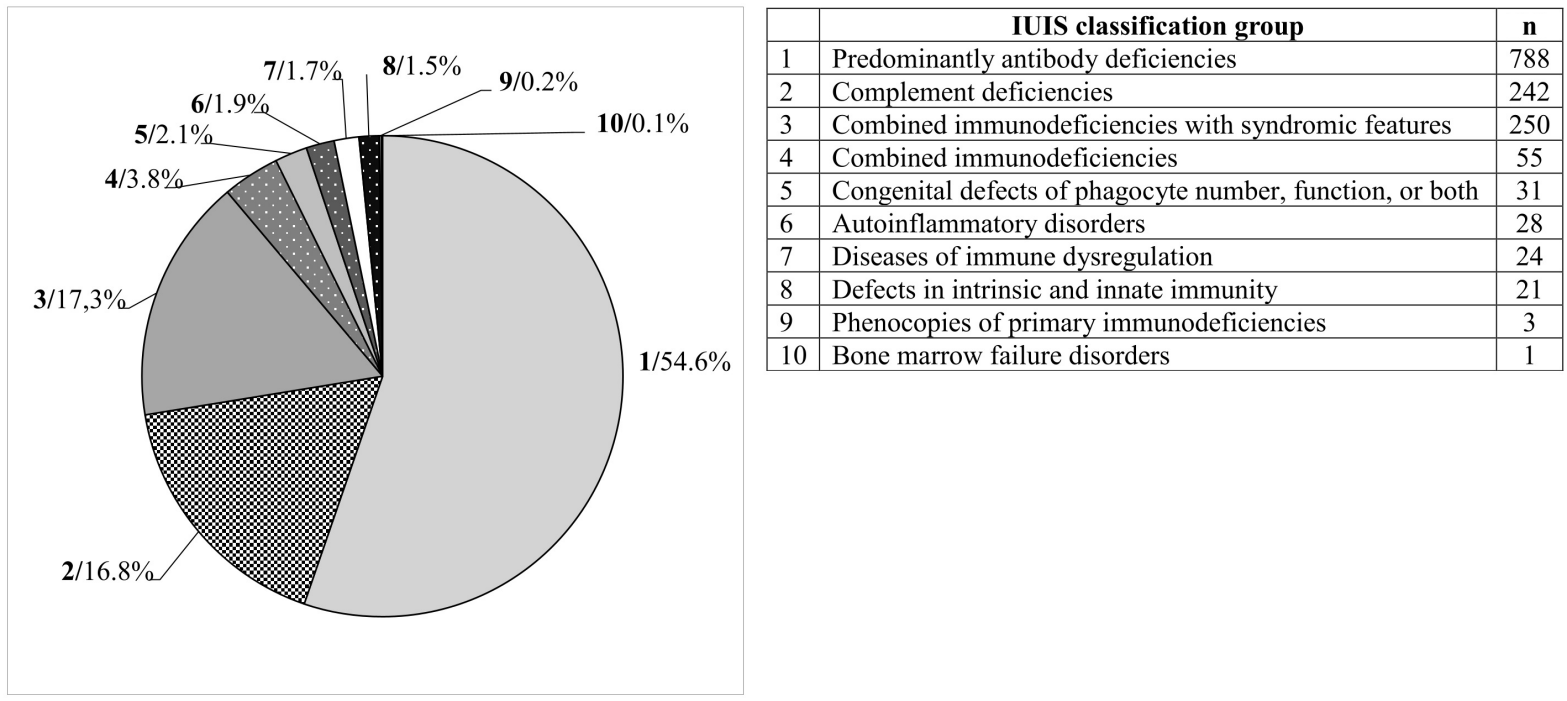
Distribution of groups of IEIs according to IUIS classification (n = 1443).

The most represented diagnostic group in the CzNR of IEIs was predominantly antibody deficiencies group (788 patients; 54.6%); see **Figure 3**. Two patients with an unknown diagnosis were excluded from further analysis. Among the 786 patients with predominantly antibody deficiencies, common variable immunodeficiency (CVID) was the most frequent diagnosis (504; 64.1%) followed by IgG subclass deficiency (85; 10.8%), clinically significant selective IgA deficiency (59; 7.5%) and X-linked agammaglobulinemia (49; 6.2%). The remaining 89 patients (11.3%) had various other, rarer antibody disorders; see **Table 1**. The second most common diagnostic group comprised patients with complement deficiencies (242; 16.8%), the majority of whom were diagnosed with hereditary angioedema (HAE) with primarily HAE C1-INH type I (182; 75.2%) and HAE C1-INH type II (35; 14.5%) along with a smaller number of HAE nC1-INH (5; 2.1%). Additionally, 15 (6.2%) patients had C2 deficiency, and 5 (2.1%) patients had other complement deficiencies including defects of the membrane attack complex (MAC); see **Table 2**. The third most common group of IEIs consisted of patients with combined immunodeficiencies with syndromic features (250; 17.3%). The most frequent diagnosis in this group was DiGeorge syndrome (182; 72.8%) followed by hyper IgE syndromes (17; 6.8%) and Wiskott-Aldrich syndrome or X-linked thrombocytopenia (13; 5.2%); see **Table 3**. Over the 13-year data collection period in the CzNR of IEIs, 55 (3.8%) patients were diagnosed with combined immunodeficiency; see **Table 4**. The most common diagnoses in this group included severe combined immunodeficiency (T^-^B^+^ SCID) affecting 17 patients (30.9%), CTLA-4 deficiency (12; 21.8%), and CD40L deficiency (9; 16.4%). The group of congenital defects of phagocyte number, function, or both comprised 31 patients (2.1%) with the most common condition being chronic granulomatous disease (CGD) (19; 61.3%), followed by neutrophil-specific granule deficiency (5; 16.1%) and Shwachman–Diamond syndrome (3; 9.7%); see **Table 5**. Other diagnostic groups of inborn errors of immunity, according to the IUIS classification, included fewer than 30 patients each in the CzNR of IEIs. A complete overview of patients in the remaining five groups is provided in **Table 6**.

**Table 1:** Czech national registry: predominantly antibody deficiencies (n = 786).

DIAGNOSIS	n (%)	FUP n (%)	DCS n (%)	L/E-FUP n (%)	Genetic diagnosis			Age at the time of diagnosis median (range)	Years of follow-up median (range)
					YES n (%)	NO n (%)	UNK n (%)		
CVID	504 (64.1)	377 (74.8)	69 (13.7)	58 (11.5)	27 (5.4)	231 (45.8)	246 (48.8)	35 (0−86)	13 (0−53)
IgGSD	85 (10.8)	61 (71.8)	7 (8.2)	17 (20.0)	3 (3.5)	38 (44.7)	44 (51.8)	42 (3−80)	11 (2−39)
sIgAD	59 (7.5)	33 (55.9)	1 (1.7)	25 (42.4)	0	32 (54.2)	27 (45.8)	29 (5−69)	10.5 (0−35)
XLA	49 (6.2)	43 (87.8)	3 (6.1)	3 (6.1)	39 (79.6)	1 (2.0)	9 (18.4)	2 (0−24)	21 (1−58)
Other	31 (3.9)	23 (74.2)	2 (6.5)	6 (19.4)	0	15 (48.4)	16 (51.6)	50 (0−83)	7 (0−25)
GDS	12 (1.5)	6 (50.0)	5 (41.7)	1 (8.3)	0	7 (58.3)	5 (41.7)	58 (44−71)	13.5 (0−19)
sIgMD	12 (1.5)	6 (50.0)	0	6 (50.0)	0	8 (66.7)	4 (33.3)	55 (22−70)	7.5 (1−30)
APDS	10 (1.3)	9 (90.0)	1 (10.0)	0	7 (70.0)	3 (30.0)	0	12 (1−47)	10.5 (6−34)
THI	8 (1.0)	3 (37.5)	0	5 (62.5)	0	3 (37.5)	5 (62.5)	1 (0−1)	2 (1−7)
AR/AD HG	6 (0.8)	6 (100.0)	0	0	3 (50.0)	0	3 (50.0)	0.5 (0−3)	2 (2−25)
AID D	3 (0.4)	3 (100.0)	0	0	3 (100.0)		0	3 (1−9)	21 (17−29)
SAD	3 (0.4)	0	0	3 (100.0)	0	2 (66.7)	1 (33.3)	16 (16−44)	3 (3−3)
NFKB2 D	2 (0.3)	2 (100.0)	0	0	2 (100.0)	0	0	2.5 (1−4)	18 (1−35)
CARD11 GOF	1 (0.1)	0	1 (100.0)	0	1 (100.0)	0	0	0	(2−2)
TRNT1 D	1 (0.1)	0	1 (100.0)	0	1 (100.0)	0	0	7 (7−7)	−
Total	786(100.0)	572 (72.8)	90 (11.4)	124 (15.8)	86 (10.9)	340 (43.3)	360 (45.8)		

n, number of patients; UNK, not reported; FUP, follow−up; DCS, deceased; L/E−FUP, lost or end of follow−up; CVID, common variable immunodeficiency; IgGSD, IgG subclass deficiency; sIgAD, selective IgA deficiency; XLA, X−linked agammaglobulinemia; THI, transient hypogammaglobulinemia of infancy; AIDD, deficiency of activation−induced cytidine deaminase; NFKB2 D, NF−kappaB2 deficiency; SAD, specific antibody deficiency with normal Ig levels and normal B cells; TRNT1D, SIFD; sideroblastic anaemia with B−cell immunodeficiency, periodic fevers, and developmental delay; AD/AR HG, autosomal recessive and dominant inborn agammaglobulinemia.

**Table 2:** Czech national registry: complement deficiencies (n = 242).

DIAGNOSIS	n (%)	FUP n (%)	DCS n (%)	L/E-FUP n (%)	Genetic diagnosis			Age at the time of diagnosis median (range)	Years of follow-up median (range)
					YES n (%)	NO n (%)	UNK n (%)		
HAE C1-INH I	182 (75.2)	169 (92.9)	6 (3.3)	7 (3.8)	128 (70.3)	29 (15.9)	25 (13.7)	20 (0−75)	15 (0−67)
HAE C1-INH II	35 (14.5)	34 (97.1)	0	1 (2.9)	30 (85.7)	1 (2.9)	4 (11.4)	18 (1−60)	13 (2−45)
HAE nC1-INH	5 (2.1)	5 (100.0)	0	0	13 (86.7)	0	2 (13.3)	33 (21−57)	3 (2−8)
C2 D	15 (6.2)	12 (80.0)	0	3 (20.0)	5 (100.0)	0	0	7 (0−60)	8 (0−30)
Other	5 (2.1)	5 (100.0)	0	0	2 (40.0)	0	3 (60.0)	33 (4−43)	4 (1−11)
Total	242 (100.0)	225 (93.0)	6 (2.5)	11 (4.5)	178 (73.6)	30 (12.4)	34 (14.0)		

n, number of patients; UNK, not reported; FUP, follow-up; DCS, deceased; L/E−FUP, lost or end of follow−up; HAE C1-INH I, hereditary angioedema type I; HAE C1-INH II, hereditary angioedema type II; HAE nC1-INH, hereditary angioedema without deficiency of C1 inhibitor; C2 D, C2 deficiency; Other, other complement deficiencies including defects of membrane attack complex.

**Table 3:** Czech national registry: combined immunodeficiencies with syndromic features (n = 250).

DIAGNOSIS	n (%)	FUP n (%)	DCS n (%)	L/E-FUP n (%)	Genetic diagnosis			Age at the time of diagnosis median (range)	Years of follow-up median (range)
					YES n (%)	NO n (%)	UNK n (%)		
DGS	182 (72.8)	175 (96.2)	2 (1.1)	5 (2.7)	156 (85.7)	1 (0.5)	25 (13.7)	0 (0−42)	18 (0−33)
HIES	17 (6.8)	16 (94.1)	1 (5.9)	0	13 (76.5)	0	4 (23.5)	13 (0−51)	8 (2−49)
WAS/XLT	13 (5.2)	12 (92.3)	1 (7.7)	0	7 (53.8)	0	6 (46.2)	1 (0−20)	13 (0−29)
Other	12 (4.8)	11 (91.7)	1 (8.3)	0	6 (50.0)	3 (25.0)	3 (25.0)	6 (0−27)	6.5 (2−25)
NBS	9 (3.6)	9 (100.0)	0	0	7 (77.8)	0	2 (22.2)	1 (0−12)	17 (5−22)
CNS	4 (1.6)	4 (100.0)	0	0	3 (75.0)	0	1 (25.0)	0 (0−15)	7.5 (3−22)
BS	4 (1.6)	4 (100.0)	0	0	4 (100.0)	0	0	6.5 (2−12)	1.5 (1−6)
AT	3 (1.2)	3 (100.0)	0	0	3 (100.0)	0	0	2 (0−2)	6 (1−10)
CHH	3 (1.2)	3 (100.0)	0	0	3 (100.0)	0	0	3 (0−17)	14 (6−14)
KS	1 (0.4)	0	0	1 (100%)	1 (100.0)	0	0	13 (13−13)	14 (14−14)
RNF168 D	1 (0.4)	1 (100.0)	0	0	1 (100.0)	0	0	5 (5−5)	11 (11−11)
SIOD	1 (0.4)	1 (100.0)	0	0	1 (100.0)	0	0	2 (2−2)	9 (9−9)
Total	250 (100.0)	239 (95.6)	5 (2.0)	6 (2.4)	205 (82.0)	4 (1.6)	41 (16.4)		

n, number of patients; UNK, not reported; FUP, follow-up; DCS, deceased; L/E−FUP, lost or end of follow−up; DGS, DiGeorge syndrome; HIES, hyper IgE syndrome; WAS/XLT, Wiskott-Aldrich syndrome, X-linked neutropenia; NBS, Nijmegen breakage syndrome; CNS, Comèl-Netherton syndrome; BS, Bloom syndrome; AT, ataxia telangiectasia; CHH, cartilage-hair hypoplasia; KS, Kabuki syndrome; RNF168 D, RIDDLE syndrome; SIOD, Schimke immuno-osseous dysplasia.

**Table 4:** Czech national registry: combined immunodeficiencies (n = 55).

DIAGNOSIS	n (%)	FUP n (%)	DCS n (%)	L/E-FUP n (%)	Genetic diagnosis			Age at the time of diagnosis median (range)	Years of follow-up median (range)
					YES n (%)	NO n (%)	UNK n (%)		
SCID T^-^B^+^	17 (30.9)	13 (76.5)	3 (17.6)	1 (5.9)	14 (82.4)	1 (5.9)	2 (11.8)	0 (0−4)	13 (0−31)
CTLA-4 D	12 (21.8)	9 (75.0)	2 (16.7)	1 (8.3)	12 (100.0)	0	0	21 (5−53)	9 (0−13)
CD40L D	9 (16.4)	9 (100.0)	0	0	5 (55.6)	0	4 (44.4)	1 (0−13)	17 (7−38)
SCID T^-^B^-^	5 (9.1)	4 (80.0)	1 (20.0)	0	4 (80.0)	0	1 (20.0)	0	9 (0−16)
Other CID	4 (7.3)	3 (75.0)	0	1 (25.0)	1 (25.0)	0	3 (75.0)	5 (0−47)	2 (1−7)
OS	2 (3.6)	1 (50.0)	1 (50.0)	0	2 (100.0)	0	0	0	8.5 (1−16)
ADA D	2 (3.6)	1 (50.0)	1 (50.0)	0	2 (100.0)	0	0	0.5 (0−1)	9 (0−18)
Artemis D	2 (3.6)	2 (100.0)	0	0	2 (100.0)	0	0	0.5 (0−1)	11.5 (10−13)
SCID (other)	1 (1.8)	0	1 (100.0)	0	0	1 (100.0)	0	0	1 (1−1)
RAG1 D	1 (1.8)	1 (100.0)	0	0	1 (100.0)	0	0	15 (15−15)	17 (17−17)
Total	55 (100.0)	43 (78.2)	9 (16.4)	3 (5.4)	43 (78.2)	2 (3.6)	10 (18.2)		

n, number of patients; UNK, not reported; FUP, follow-up; DCS, deceased; L/E−FUP, lost or end of follow−up; SCID, severe combined immunodeficiency; CTLA-4 D, cytotoxic T-lymphocyte associated protein 4 deficiency; CD40L D, CD40 ligand deficiency; CID, combined immunodeficiency; OS, Omenn syndrome; ADA D, adenosine deaminase deficiency; Artemis D, Artemis deficiency; RAG1 D, RAG1 deficiency.

**Table 5:** Czech national registry: congenital defects of phagocyte number, function, or both (n = 31).

DIAGNOSIS	n (%)	FUP n (%)	DCS n (%)	L/E-FUP n (%)	Genetic diagnosis			Age at the time of diagnosis median (range)	Years of follow-up median (range)
					YES n (%)	NO n (%)	UNK n (%)		
Autoinflammatory disorders									
CGD	19 (61.3)	18 (94.7)	1 (5.3)	0	9 (47.4)	1 (5.3)	9 (47.4)	2 (0−20)	19 (1−53.0)
SGD	5 (16.1)	4 (80.0)	0	1 (20.0)	0	1 (20.0)	4 (80.0)	9 (4−51)	13 (8−14.0)
SDS	3 (9.7)	0	0	3 (100.0)	1 (33.3)	0	2 (66.7)	8 (7−11)	7 (7−7)
MPOD	2 (6.5)	1 (50.0)	0	1 (50.0)	0	2 (100.0)	0	38.5 (26−51)	7 (1−13)
LAD	1 (3.2)	1 (100.0)	0	0	0	0	1 (100.0)	6 (6−6)	31 (31−31)
GATA2D	1 (3.2)	1 (100.0)	0	0	1 (100.0)	0	0	9 (9−9)	7 (7−7)
Total	31 (100.0)	25 (80.7)	1 (3.2)	5 (16.1)	11 (35.5)	4 (12.9)	16 (51.6)		

n, number of patients; UNK, not reported; FUP, follow-up; DCS, deceased; L/E−FUP, lost or end of follow−up; CGD, chronic granulomatous disease; SGD, neutrophil-specific granule deficiency; SDS, Shwachman-Diamond syndrome; MPOD, myeloperoxidase deficiency; LAD, leukocyte adhesion deficiency; GATA2D, GATA 2 deficiency.

**Table 6:** Czech national registry: autoinflammatory disorders (n = 28), diseases of immune dysregulation (n = 24), defects in intrinsic and innate immunity (n = 21), phenocopies of PID (n = 3), bone marrow failure disorders (n = 1).

DIAGNOSIS	n (%)	FUP n (%)	DCS n (%)	L/E-FUP n (%)	Genetic diagnosis			Age at the time of diagnosis median (range)	Years of follow-up median (range)
					YES n (%)	NO n (%)	UNK n (%)		
Autoinflammatory disorders									
FMF	14 (50,0)	13 (92.9)	0	1 (7.1)	13 (92.9)	0	1 (7.1)	39 (1−58)	12 (1−20)
HIDS	5 (17,9)	5 (100.0)	0	0	4 (80.0)	0	1 (20.0)	10 (2−48)	16 (7−18)
SchS	4 (14,3)	4 (100.0)	0	0	2 (50.0)	1 (25.0)	1 (25.0)	14 (2−46)	3 (2−25)
TRAPS	3 (10,7)	3 (100.0)	0	0	0	0	3 (100.0)	42 (41−64)	7 (7−19)
Other	2 (7,1)	2 (100.0)	0	0	2 (100.0)	0	0	35 (9−61)	14 (12−17)
Total	28 (100.0)	27 (96.4)	0	1 (3.6)	21 (75.0)	1 (3.6)	6 (21.4)		
Diseases of immune dysregulation									
HLH	11 (45,8)	10 (90.9)	1 (9.1)	0	6 (54.5)	0	5 (45.5)	0 (0−9)	13 (0−23)
Other	4 (16,7)	4 (100.0)	0	0	3 (75.0)	0	1 (25.0)	7 (3−11)	6 (5−7)
XLP-2	4 (16,7)	3 (75.0))	1 (25.0)	0	3 (75.0)	0	1 (25.0)	1.5 (1−31)	6 (0−9)
XLP	2 (8,3)	2 (100.0)	0	0	1 (50.0)	0	1 (50.0)	5.5 (4−7)	25 (17−33)
CHS	1 (4,2)	1 (100.0)	0	0	0	0	1 (100.0)	1 (1−1)	19 (19−19)
IPEX	1 (4,2)	1 (100.0)	0	0	1 (100.0)	0	0	0 (0−0)	10 (10−10)
STAT3 GOF	1 (4,2)	0	1 (100.0)	0	1 (100.0)	0	0	6 (6−6)	1 (1−1)
Total	24 (100.0)	21 (87.5)	3 (12.5)	0	15 (62.5)	0	9 (37.5)		
Defects in intrinsic and innate immunity									
CMC	16 (76,2)	13 (81.3)	3 (18.8)	0	14 (87.5)	0	2 (12.5)	12.5 (1−50)	6 (0−14)
Other	5 (23,8)	5 (100.0)	0	0	5 (100.0)	0	0	9 (2−43)	4 (1−8)
Total	21 (100.0)	18 (85.7)	3 (14.3)	0	19 (90.5)	0	2 (9.5)		
Phenocopies of PID									
AAE	3 (100.0)	2 (66.7)	1 (33.3)	0	1 (33.3)	0	2 (66.7)	56 (48−76)	7 (4−8)
Bone marrow failure disorders									
SAMD9	1 (100.0)	0	1 (100.0)	0	1 (100.0)	0	0	5 (5−5)	0 (0−0)

n, number of patients; UNK, not reported; FUP, follow-up; DCS, deceased; L/E−FUP, lost or end of follow−up; FMF, Familial Mediterranean fever; HIDS, Hyper-IgD syndrome; SchS, Schnitzler syndrome; TRAPS, Tumor-necrosis factor receptor type 1 Associated Periodic Syndrome; HLH, Familial Hemophagocytic Lymphohistiocytosis; XLP-2, X-linked lymphoproliferative disease type 2; XLP, X-linked lymphoproliferative disease; CHS, Chédiak-Higashi Syndrome; IPEX, Immune dysregulation, polyendocrinopathy, enteropathy X-linked syndrome; STAT 3, Signal transducer and activator of transcription 3 gain of function mutation; CMC, Chronic Mucocutaneous Candidiasis; AAE, acquired angioedema; SAMD9, sterile alpha motif domain containing 9 gene mutation.

### 3.3 Geographic distribution of patients in the regions of the Czech Republic

**Figure 4:**
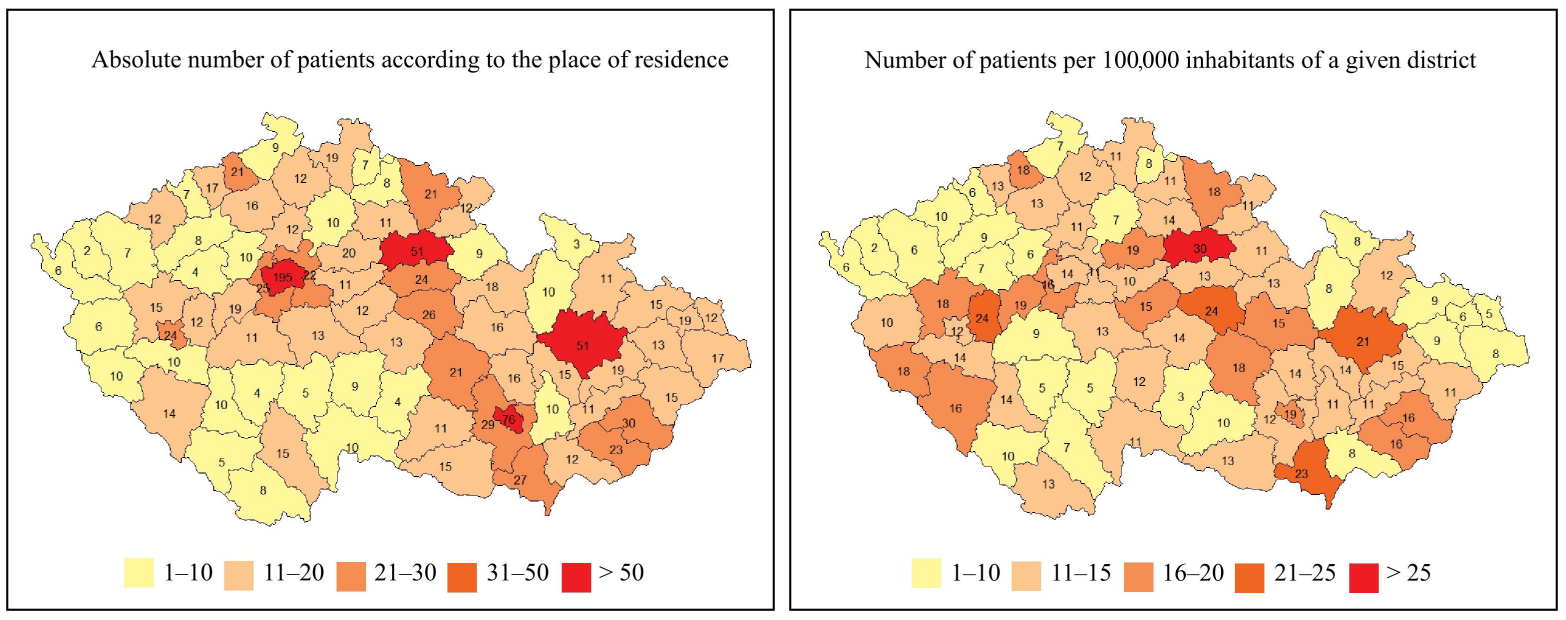
Geographic distributions of IEIs patients in the 76 regions of the Czech Republic (n = 1384).

**Figure 5:**
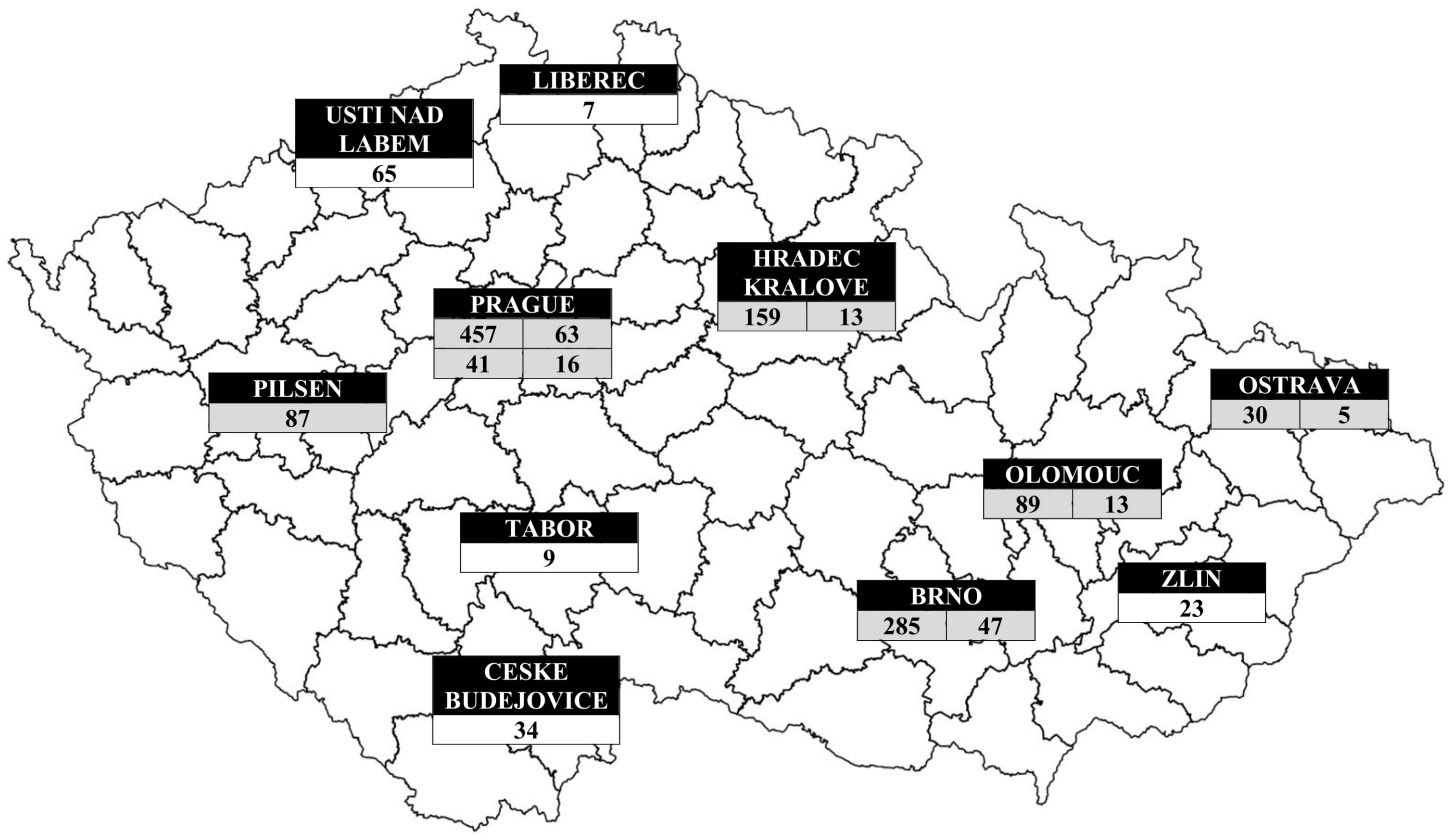
Geographic distributions of IEIs patients according to centres. Grey (centres in university hospitals), white (centres in private clinics or public hospitals): Prague → Department of Immunology, Second Faculty of Medicine and University Hospital in Motol, Charles University (457 patients), Institute of Immunology and Microbiology of the First Faculty of Medicine and General University Hospital (63 patients), Department of Clinical Immunology and Allergology, Institute for Clinical and Experimental Medicine (41 patients) and Department of Allergology and Clinical Immunology, Faculty Hospital Kralovske Vinohrady (16 patients); South Bohemian Region → Centre for Clinical Immunology, Hospital Ceske Budejovice (34 patients), Kasmed Ltd. in Tabor (9 patients); Plzen Region → Department of Allergology and Clinical Immunology, University Hospital Pilsen (87 patients); Usti nad Labem Region → Department of Clinical Immunology and Allergology, Masaryk Hospital Usti nad Labem (65 patients); Liberec Region → Department of Clinical Microbiology and Immunology, Liberec Regional Hospital (7 patients); Hradec Kralove Region → Institute of Clinical Immunology and Allergy (159 patients) and Department of Pediatrics (13 patients) of University Hospital and Faculty of Medicine in Hradec Kralove, Charles University; South Moravian Region → Department of Clinical Immunology and Allergology of St. Anne´s University Hospital in Brno (285 patients), Department of Pediatrics of University Hospital Brno (47 patients); Olomouc Region → Department of Allergology and Clinical Immunology, University Hospital Olomouc (89 patients) and Department of Pediatrics, Faculty of Medicine and Dentistry, Palacky University (13 patients); Zlin Region → Department of Pulmonary Medicine, Tomas Bata Regional Hospital Zlin (23 patients) and Moravian-Silesian Region → Department of Immunology and Allergy, Public Health Institute Ostrava (30 patients), Department of Allergology and Clinical Immunology, Faculty Hospital Ostrava (5 patients).

The geographical distribution of patients with IEIs based on their place of residence is shown in **Figure 4**. As expected, the highest concentration of patients with IEIs was found in the capital city of Prague (nearly 1.4 million inhabitants, approximately 13.0% of the Czech population), as well as in other large regional or district cities. The geographical distribution of IEIs centers across the Czech Republic, along with the number of patients being followed and treated at each center, is presented in **Figure 5**.

### 3.4 Treatment

#### 3.4.1 Immunoglobulin replacement therapy

Out of a total of 1,173 continuously monitored patients in the Czech national registry, 538 (45.9%) received immunoglobulin replacement therapy (IRT); see **Table 7**. The majority of these patients (504; 93.7%) belonged to the group of predominantly antibody deficiencies, followed by combined immunodeficiencies with syndromic features (21; 3.9%) and combined immunodeficiencies (7; 1.3%). At the time of data analysis, the most common route of IRT administration was subcutaneous immunoglobulin (SCIG) therapy (388; 72.1%), which included both conventional SCIG treatment (223; 57.5%) and facilitated SCIG treatment using hyaluronidase (165; 42.5%). Intravenous immunoglobulin replacement therapy (IVIG) was administered to 146 (27.1%) patients, while 4 (0.7%) patients had previously received intramuscular immunoglobulin (IMIG) therapy.

**Table 7:** Czech national registry: data of immunoglobulin replacement therapy in patients with IEIs.

IUIS classification groups	n	IVIG	SCIG	fSCIG	IMIG
Predominantly antibody deficiencies	504	137 (27.2%)	208 (41.3%)	156 (31.0%)	3 (0.6%)
Combined immunodeficiencies with syndromic features	21	8 (38.1%)	10 (47.6%)	2 (9.5%)	1 (4.8%)
Combined immunodeficiencies	7	1 (14.3%)	3 (42.9%)	3 (42.9%)	0
Defects in intrinsic and innate immunity	3	0	2 (66.7%)	1 (33.3%)	0
Diseases of immune dysregulation	2	0	0	2 (100.0%)	0
Autoinflammatory disorders	1	0	0	1 (100.0%)	0

n, number of patients; DP, deceased patients; IVIG, intravenous immunoglobulin replacement therapy; SCIG, subcutaneous immunoglobulin replacement therapy; fSCIG, subcutaneous immunoglobulin replacement therapy facilitated by hyaluronidase; IMIG, immunoglobulin for intramuscular administration.

#### 3.4.2 Hematopoietic stem cell transplantation

The Czech National Registry includes 81 patients with IEIs who underwent HSCT, of whom 14 subsequently died; see **Table 8**. These figures include all patients who were alive at any point during the registry’s existence. In total, 94 patients with IEIs have undergone HSCT at the only transplantation center for IEI patients in the Czech Republic (Faculty Hospital Motol, Prague) since the introduction of this treatment in 1994, with 22 reported deaths. During the period from April 2014 to April 2025, a total of 37 patients received transplants at the center, and 6 of them died.

**Table 8:** Czech national registry: data of performed hematopoietic stem cell transplantation (n = 81).

IUIS classification groups	Hematopoietic stem cell transplantation (HSCT)		
	Underwent HSCT	Alive	Died
Predominantly antibody deficiencies	3	1	2
Complement deficiencies	0	−	−
Combined immunodeficiencies with syndromic features	14	13	1
Combined immunodeficiencies	34	28	6
Congenital defects of phagocyte number, function, or both	10	9	1
Autoinflammatory disorders	0	−	−
Diseases of immune dysregulation	19	16	3
Defects in intrinsic and innate immunity	1	1	0
Phenocopies of PID	0	−	−
Bone marrow failure disorders	1	0	1

### 3.5 Deceased patients

**Figure 6:**
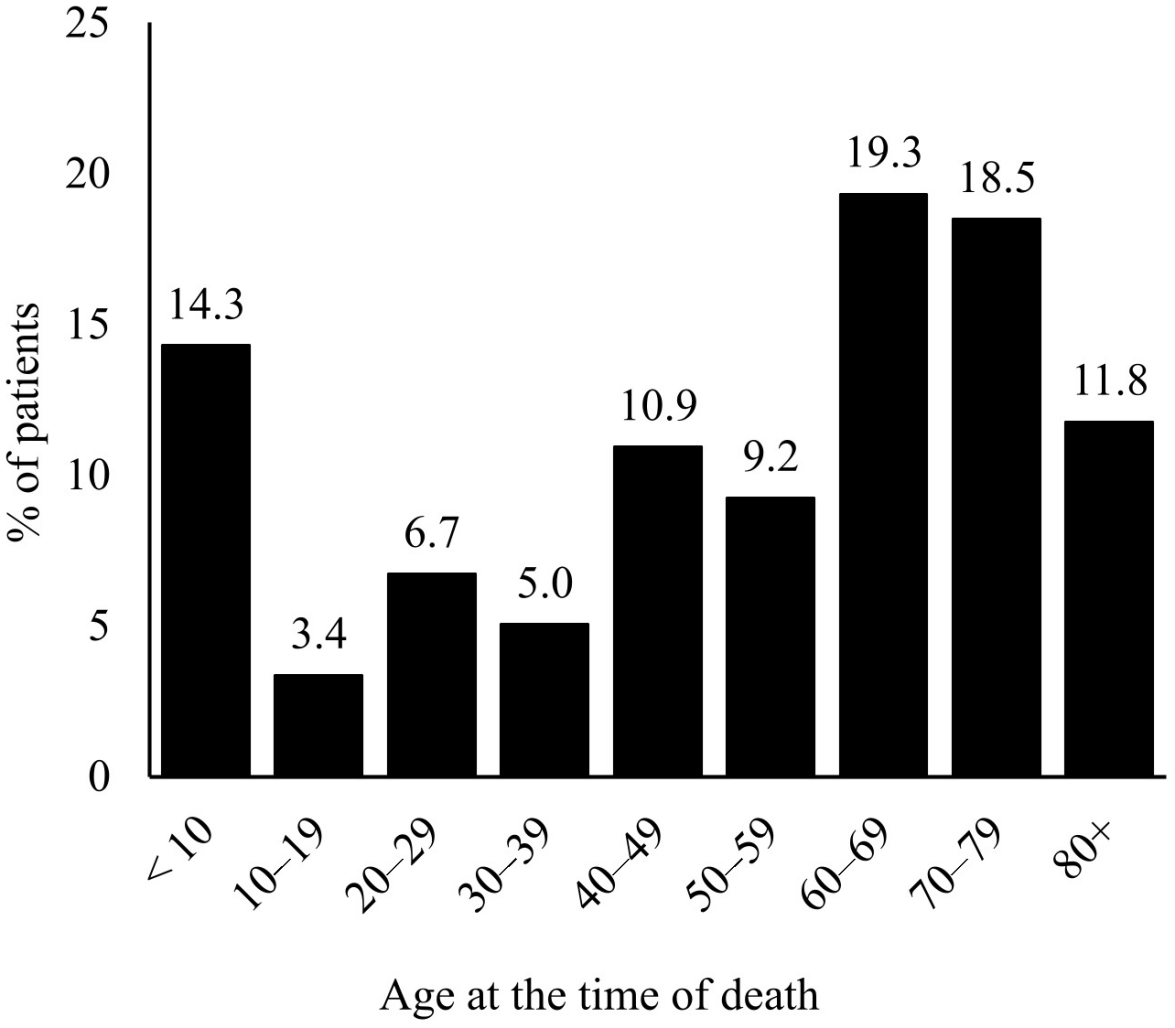
Age distribution of deceased patients with IEIs from 2012−2025.

A total of 119 patients with IEIs died during the 13-year observation period. Of these, 66 (55.5%) were males and 53 (44.5%) were females. The average age at the time of death was 50.7 ± 26.8 years (median 59.5 years; range 0–88); see **Figure 6**. The mean follow-up time from diagnosis to death was 13.8 ± 11.3 years (median 12 years; range 0–58). The majority of deceased patients belonged to the largest diagnostic group of predominantly antibody deficiencies (90; 75.6% of all deceased patients), followed by combined immunodeficiencies (9; 7.6%) and complement deficiencies (6; 5.0%). However, the highest mortality rates relative to group size (based on the IUIS classification) were observed in patients with bone marrow failure disorders (1; 100.0%), phenocopies of primary immunodeficiencies (1; 33.3%), and combined immunodeficiencies (9; 16.4%); see **Table 9**. In most cases, the cause of death was not specified. The most common cause of death was infectious complications (30; 25.2%), followed by malignancies (14; 11.8%) and cardiac failure (14; 11.8%). Less frequent causes of death included respiratory failure (7; 5.9%), multiorgan failure (6; 5.0%), liver failure (5; 4.2%), stroke (3; 2.5%), and complications following HSCT (2; 1.7%). Other reported causes of death included acute pancreatitis (2; 1,7%), kachexia (2; 1,7%), complications following a subtrochanteric fracture of the right femur (1; 0.8%), suicide (1; 0.8%), chronic rejection of the transplant (1; 0.8%), aneurysm rupture (1; 0.8%), or asphyxia due to a laryngeal attack of HAE (1; 0.8%).

**Table 9:** Czech national registry: number of deceased patients (n = 119) with IEIs from 2012−2025.

IUIS classification groups	n of DP	Cause of death										% of DP out of all DP	% of DP out of all IEIs patients in the IUIS group
		*Infection*	*Malignancy*	*Cardiac failure*	*Respiratory failure*	*Multiorgan failure*	*Liver failure*	*Stroke*	*Complication HSCT*	*Other*	*Unknown*		
Predominantly antibody deficiencies	90	26	13	10	6	3	5	2	0	5	20	75.6	11.4
Combined immunodeficiencies	9	2	0	1	0	1	0	0	1	1	3	7.6	16.4
Complement deficiencies	6	0	0	0	0	0	0	0	0	1	5	5.0	2.5
Combined immunodeficiencies with syndromic features	5	1	0	2	1	0	0	0	0	1	0	4.2	2.0
Diseases of immune dysregulation	3	1	0	1	0	1	0	0	0	0	0	2.5	12.5
Defects in intrinsic and innate immunity	3	0	0	0	0	1	0	0	1	1	0	2.5	14.3
Congenital defects of phagocyte number, function, or both	1	0	0	0	0	0	0	1	0	0	0	0.8	3.2
Bone marrow failure disorders	1	0	0	0	0	0	0	0	0	0	1	0.8	100.0
Phenocopies of primary immunodeficiencies	1	0	1	0	0	0	0	0	0	0	0	0.8	33.3
Summary: causes of death	119	30	14	14	7	6	5	3	2	9	29	−	−

n, number of patients; DP, deceased patients.

## 4 Discussion

Over the past few decades, rapid advancements in the understanding of IEIs have led to a growing number of recognized disorders, driven in part by progress in immunological laboratory diagnostics and genetic testing. Patient registries of IEIs represent another essential tool for collecting data on the epidemiology, diagnostic procedures, clinical course, and treatment of affected individuals. A key advantage of these registries is that they contain real-world data from routine clinical practice, thereby enhancing our understanding of the clinical and laboratory spectrum, as well as treatment responses.

Large international IEIs registries provide the added benefit of including data from broader and more diverse populations. These larger datasets enhance the statistical power of analyses, enabling the identification of patterns, correlations, and potential therapies that might remain hidden in smaller studies. Conversely, national registries allow us to highlight differences in the prevalence of specific diagnoses across populations with distinct genetic, environmental, and social backgrounds. Understanding the geographic distribution of patients with IEIs is also important for planning healthcare services.

Although congenital immune system disorders are generally considered rare, their global prevalence is likely underestimated. A recent systematic review by Abolhassani et al. compiled data from national reports of 80 countries and major international registries (22). The authors identified over 120,000 unique IEIs patients enrolled in registries at the time. The study confirmed the absence of comprehensive prevalence data for many countries and continents. For example, Asia, home to approximately 4.7 billion people or 59.0% of the global population, provided data through only 18 national registries (out of 49 countries), covering just 15,939 IEIs patients. In Africa, with a population of 1.2 billion (15.0% of the global population), only 7 out of 54 countries (12.9%) had published IEIs registry data (22). Oceania, with 43 million people across 15 countries, had data from just 2 countries (13.2%) (22). In contrast, Europe has much better availability of registry data, largely thanks to multiple national and international efforts. The first European national IEI registry was published in 1983 by Italian researchers (9), marking a milestone in systematic data collection on these rare disorders. The first international registry of PID was established by the European Society for Immunodeficiencies (ESID) in 1994 (21). This registry aimed to collect data on patients with primary immunodeficiency disorders across Europe. Since direct database access was initially unavailable to contributing centers, a new ESID online registry was launched in 2004, aiming to serve as a central registry for Europe and other participating countries (17). At that time, no unified disease classification existed. To improve quality assurance and data utility, the registry underwent major restructuring in 2014 (23). In 2019, the ESID registry’s working definitions for clinical diagnosis of IEIs were published, enabling classification even in cases without a known genetic cause (21). According to 2019 data, the ESID registry included information on over 25,000 patients (21), but the last comprehensive report was published in 2014 (23). Back then, IEI prevalence in Europe was estimated at between 1 in 16,000 to 1 in 50,000 (23), although experts suggest the true prevalence may be significantly higher (17).

The Czech Republic, a relatively small country in Central Europe with nearly 11 million inhabitants, has a predominantly Czech population and a low rate of consanguinity. Since the launch of the Czech online registry platform thirteen years ago, all immunology centers caring for IEIs patients have actively contributed to the database. The number of registered patients has steadily increased from 410 in 2012 to 1,443 in 2025. As such, the Czech national registry likely provides a realistic estimate of the number of patients receiving proper diagnostic and therapeutic care for IEI in the country. One of the key strengths of the National registry of IEIs is that patients with IEIs in the Czech Republic are monitored in specialized centers, all of which collaborate effectively in entering data into the registry and also provide relatively even coverage across all geographic regions of the country. This allows us to assume that the majority of patients with IEI are included in the registry. Furthermore, genetic testing is relatively accessible in the Czech Republic and is carried out in two main genetic laboratories located in Prague and Brno. A limitation of the national IEI registry is the insufficient data collection in certain areas, particularly regarding autoimmune and malignant complications in patients with IEI, which are among the core clinical manifestations in some of these individuals. The registry does not collect data on diagnostic delay, but only on the age at the time of diagnosis. Furthermore, the causes of death recorded in the registry may not necessarily reflect the actual causes. The registry does not include information on whether an autopsy was performed in individual cases. Therefore, the cause of death can only be estimated by general practitioner or the attending physician and are rather indicative. Moreover, patients with various types of diagnoses falling under the group of bone marrow failure disorders are likely not all followed up at immunology centers and therefore are not included in the national registry. Consequently, the reported number of patients in this group is likely an underestimation of the true prevalence in the Czech Republic.

This study is the first to report on the prevalence of IEI in the Czech Republic, which is approximately 1 in 8,000 inhabitants, roughly twice the lower end of the previously estimated European prevalence range (23). When comparing the number of patients in IEI registries from the five most populous European countries (Germany, the United Kingdom, France, Italy, and Spain), for which the national registry data were published, the CzNR of IEIs includes approximately 2−3 times more IEIs patients. This fact is most likely due to the well-organized care for these patients, who are monitored in specialized centers that more or less evenly cover the entire territory of the country, rather than a genuinely higher prevalence of patients with congenital immune system disorders in the Czech Republic. While the total number of registered patients in the Czech national registry was 1,443 patients out of 11 million inhabitants (1:8,000), the United Kingdom’s registry had recruited 4,758 patients in more than 66 million of inhabitants in 2017 (1:14,000) (16), the French national registry comprised a total of 3,083 patients in approximately 61 million inhabitants in 2010 (1:20,000) (8), the Italian database contained a total of 3,352 pediatric and adult patients out of nearly 60 million inhabitants in 2019 (1:18,000) (24), and the Spanish national registry referred data about 2,050 registered patients from nearly 40.5 million inhabitants in 2011 (1:20,000) (25). Germany is an exception in that, despite its larger population, the absolute number of registered patients is similar to that in the Czech Republic. A total number of 1,368 patients were reported in the registry out of more than 80.5 million inhabitants in 2013 (1:59,000) (7). Across all countries, predominantly antibody deficiencies made up the largest proportion of diagnoses, namely the Czech Republic 786 (54.3%), Germany 858 patients (62.7%) (7), United Kingdom 2,589 (60.0%) (16), France (8), Italy (24), and Spain 1,403 (68.4%) (25). This was followed by complement deficiencies, combined immunodeficiencies, or combined immunodeficiencies with syndromic features in the CzNR. Although most national registries do not report as many patients with complement deficiencies, our data are consistent with, for example, the registry in Ireland (6), where complement disorders also rank second in terms of patient numbers. This is due to the fact that care for HAE patients is centralized in four centers, which are responsible for entering data into the registry in a consistent and reliable manner. In summary, the distribution of IEIs patients into different groups according to their IUIS classification roughly corresponds to the various countries being compared.

## 5 Conclusion

Patient registries provide important information on the epidemiology and outcomes of patients with various diagnoses, which is especially valuable in the case of rare diseases. The online platform of the CzNR of IEIs was established in 2012. As of 2025, the total number of 1,443 patients had been registered in the CzNR, out of a population of 11 million, resulting in a calculated prevalence of 13.2 cases per 100,000 inhabitants. The most represented group of patients in the registry is those with predominantly antibody deficiencies, with common variable immunodeficiency (CVID) being the most frequent diagnosis. Data collected from national and international IEI registries contribute to a comprehensive understanding of clinical manifestations, complications, diagnostic procedures, and treatment strategies by gathering detailed clinical, laboratory, and genetic information on affected individuals.

## Data Availability

The original contributions presented in the study are included in the article/supplementary material. Further inquiries can be directed to the corresponding author.
